# The Involvement of *YNR069C* in Protein Synthesis in the Baker’s Yeast, *Saccharomyces cerevisiae*

**DOI:** 10.3390/biology13030138

**Published:** 2024-02-22

**Authors:** Sarah Takallou, Maryam Hajikarimlou, Mustafa Al-gafari, Jiashu Wang, Thomas David Daniel Kazmirchuk, Kamaledin B. Said, Bahram Samanfar, Ashkan Golshani

**Affiliations:** 1 Ottawa Institute of Systems Biology, University of Ottawa, Ottawa, ON K1N 6N5, Canada; saratakalloo@cmail.carleton.ca (S.T.); maryamhajikarimlou@cmail.carleton.ca (M.H.); mustafaalgafari@cmail.carleton.ca (M.A.-g.); jiashuwang@cmail.carleton.ca (J.W.); tomkazmirchuk@cmail.carleton.ca (T.D.D.K.); bahram.samanfar@agr.gc.ca (B.S.); 2 Department of Biology, Carleton University, Ottawa, ON K1S 5B6, Canada; 3 Department of Pathology and Microbiology, College of Medicine, University of Hail, Hail 55476, Saudi Arabia; 4 Agriculture and Agri-Food Canada, Ottawa Research and Development Centre (ORDC), Ottawa, ON K1A 0C6, Canada

**Keywords:** gene expression, codon, protein synthesis, premature stop codon, yeast

## Abstract

**Simple Summary:**

Preserving the integrity of protein biosynthesis involves maintaining a delicate equilibrium between translation accuracy and speed. In the current study we observed that deletion of *YNR069C* resulted in a notable increase in readthrough at premature stop codons, suggesting its participation in the process of translation. Moreover, the absence of *YNR069C* led to an acceleration of the translation rate. These observations indicate that *YNR069C* may play a role in translation integrity. Our comprehensive genetic interaction analysis reinforces this idea, establishing a clear link between *YNR069C* and the regulation of translation. These findings contribute to our understanding of how cells may control gene expression.

**Abstract:**

Maintaining translation fidelity is a critical step within the process of gene expression. It requires the involvement of numerous regulatory elements to ensure the synthesis of functional proteins. The efficient termination of protein synthesis can play a crucial role in preserving this fidelity. Here, we report on investigating a protein of unknown function, *YNR069C* (also known as *BSC5*), for its activity in the process of translation. We observed a significant increase in the bypass of premature stop codons upon the deletion of *YNR069C*. Interestingly, the genomic arrangement of this ORF suggests a compatible mode of expression reliant on translational readthrough, incorporating the neighboring open reading frame. We also showed that the deletion of *YNR069C* results in an increase in the rate of translation. Based on our results, we propose that *YNR069C* may play a role in translation fidelity, impacting the overall quantity and quality of translation. Our genetic interaction analysis supports our hypothesis, associating the role of *YNR069C* to the regulation of protein synthesis.

## 1. Introduction

Translation termination occurs when one of the three stop codons (UAA, UAG, and UGA) is encountered by the translation machinery in the ribosomal A-site. In eukaryotes, two release factors, eRF1 and eRF3, are involved in the process of termination. When eRF1, in association with eRF3, recognizes a stop codon, it occupies the A-site of the ribosome by mimicking the tRNA structure [1]. Subsequently, it induces the hydrolysis of peptidyl-tRNA by the ribosome, resulting in the release of the nascent peptide. eRF3, which forms a heterodimer with eRF1, functioning as a GTPase, binds to the ribosome and actively facilitates the dissociation of eRF1, contributing to the completion of the termination process [2,3].

Stop codon readthrough occurs when a near cognate tRNA binds to a stop codon instead of eRF1, resulting in the continuation of translation elongation until the subsequent in-frame stop codon [4]. Decreased cellular levels of eRF1 are shown to be associated with an increased occurrence of stop codon bypass [5].

In certain cases, stop codon readthrough can serve as a regulatory mechanism in gene expression, enabling the production of different polypeptides from a single gene. This is a common regulatory mechanism in RNA viruses, facilitating the expansion of the genetic information of their compact genomes [6,7]. In both yeast and bacteria, increased translational errors, or mistranslations, have been found to introduce certain advantageous phenotypes [8,9]. For example, in *Candida albicans*, such mistranslations can lead to phenotypic diversity, resulting in different cell morphotypes [10]. In *Saccharomyces cerevisiae*, it is thought to offer a transient survival advantage in certain conditions, enhancing cell adaptability [11].

In mammals, reduced translational accuracy, particularly at the stop codons, can be advantageous for cells, leading to the readthrough of premature stop codons (PTC), ultimately benefiting the synthesis of full-length proteins [12]. This is employed as a treatment strategy in certain cases. Studies have shown that aminoglycoside antibiotics can reduce the accuracy of translation by binding to the ribosome and interfering with proofreading mechanisms. This can lead to a readthrough of PTCs and the synthesis of full-length proteins, which can be beneficial in certain conditions. For example, in cystic fibrosis, aminoglycosides have been shown to increase the production of functional CFTR protein by promoting the readthrough of premature stop codons [13].

In a recent study, we investigated genes involved in PTC readthrough in yeast. We identified several candidates through large-scale screening when their deletion increased the production of full-length protein from an mRNA with a premature stop codon [14]. In the current study, we repeated the screen using a plasmid that carries a UAA premature stop codon within the *β-galactosidase* reporter gene. This led us to identify the gene *YNR069C*, also known as *BSC5* (Bypass of Stop Codon 5), that was not detected in our previous screens and observed increased *β-galactosidase* production upon its deletion.

This gene codes for a protein of unknown function, making it an interesting target for further investigation. Of interest, *YNR069C* is reported to carry a premature stop codon, allowing it to encode two possible products: a shorter protein, which is the result of termination at the premature stop codon, and a full-length protein, which is the result of a bypass of the premature stop codon [15]. *YNR069C* is a member of the yeast α-arrestin protein family, which is known for its structural and functional similarities to human α-arrestins. These proteins primarily act as adaptor proteins, facilitating the Rsp5 ligase-mediated ubiquitination of plasma membrane proteins [16]. In this study, we demonstrated that *YNR069C* plays a role in translation fidelity.

## 2. Materials and Methods

### 2.1. Strains and Media

The study utilized yeast, *S. cerevisiae,* MATa strain BY4741 (orfΔ::kanMAX4 his3Δ1 leu2Δ0 met15Δ0 ura3Δ0) and MATα strain, BY7092 (can1Δ::STE2pr-Sp_his5 lyp1Δ his3Δ1 leu21Δ0 ura3Δ0 met15Δ0). Mutant strains were obtained from the yeast non-essential gene knockout mutant library. This library was also employed to produce double mutants for Synthetic Genetic Analysis (SGA) [17]. Plasmids for overexpressing candidate genes were derived from the yeast overexpression collection [18]. The mutant strains for *YNR069C* used in the follow-up investigation were generated through homologous recombination by replacing the corresponding ORF with the nourseothricin sulfate (clonNAT) resistance gene in the WT background strain (BY7092).

Yeast was cultured in standard rich YP media (1% Yeast extract, 2% Peptone) and Synthetic Complete (SC) media with selective amino acids (0.67% Yeast nitrogen base w/o amino acids, 0.2% Dropout mix). To each of these media, we added either 2% D-glucose, making YPD media or 2% galactose, yielding YPgal media. *E. coli* was grown in LB (Lysogeny broth). Two percent agar was used for all solid media [19,20].

### 2.2. Plasmids

The constructs, pUKC817, pUKC818, and pUKC819, carrying premature stop codons UAA, UGA, and UAG, respectively, within the *β-galactosidase* under the transcriptional control of *PGK1* promoter, were employed in this study [21]. pUKC815 serves as a control without any premature stop codon [22].

The translation rate was evaluated using plasmids p416, which contain a Gal-inducible *β-galactosidase* [23]. The pAG25 plasmid served as a DNA template in PCR reactions to amplify the clonNAT resistance gene marker [24]. Each plasmid contained an ampicillin resistance gene and a *URA3* marker gene serving as a selection marker in *E. coli* DH5α and yeast, respectively.

### 2.3. β-Galactosidase Assay

A large-scale *β-galactosidase* lift assay using the X-gal compound was performed to determine the translation activity of the *β-galactosidase* reporter gene, as described [25]. A quantitative *β-galactosidase* assay was conducted using ONPG (O-nitrophenyl-α-D-galactopyranoside) as described [21,26].

### 2.4. qRT-PCR

Total RNA extraction was performed using the Qiagen^®^ (Hilden, Germany) RNeasy Mini Kit. Complementary DNA (cDNA) was synthesized using the iScript Select cDNA Synthesis Kit from Bio-Rad^®^ (Hercules, CA, USA) following the manufacturer’s instructions. The synthesized cDNA served as a template for quantitative PCR. To ensure the absence of contaminating genomic or plasmid DNA, no-RT controls were included in the assays. These controls consisted of reactions without the reverse transcriptase enzyme, confirming that the amplification signals were derived exclusively from RNA templates. The qPCR was conducted utilizing Bio-Rad^®^ (Hercules, CA, USA) iQ SYBR Green Supermix and the CFX Connect Real-Time System (Bio-Rad^®^, Hercules, CA, USA), following the manufacturer’s instructions. Amplification efficiencies for each primer pair were determined using a standard curve method. Serial dilutions of cDNA were used to generate standard curves, and the efficiency was calculated based on the slope of the linear regression plot, ensuring reliable quantification. *PGK1* was utilized as an internal control. The primers used are as follows: *β-galactosidase* Forward: TTGAAAATGGTCTGCTGCTG; Reverse: TATTGGCTTCATCCACCACA; *PGK1* Forward: ATGTCTTTATCTTCAAAGTT; Reverse: TTATTTCTTTTCGGATAAGA. The analysis of qPCR data was conducted using the comparative CT (ΔΔCT) method. We normalized the expression levels of our genes of interest to the expression of a reference gene, ensuring the data were standardized for variations in cDNA quantity and quality. This normalization allows for the comparison of expression levels across different samples [27,28].

### 2.5. Genetic Interaction Analysis

In the process of conducting a Synthetic Genetic Array (SGA) analysis, we utilized a systematic approach to generate double mutant strains in *S. cerevisiae*. Initially, a MATα strain carrying a query mutation (*YNR069C*), which was replaced by a nourseothricin resistance gene, was crossed with a MATa deletion array. In this array, each gene deletion was marked kanMX, conferring resistance to geneticin [29]. Following the mating, the resultant zygotes were selected on a medium containing both nourseothricin and geneticin to ensure the presence of both mutations. The heterozygous diploids from this selection were then transferred to a medium with reduced nutrition to induce sporulation and facilitate the formation of haploid progeny. In the final selection stage, we isolated haploids of the MATa type, ensuring they carried both the query mutation and the gene deletions from the deletion array. This systematic approach enabled us to successfully generate double mutants for subsequent analyses, effectively combining the genetic backgrounds of the parental strains [30]. We utilized the SGAtools (http://sgatools.ccbr.utoronto.ca), an automated software tool, for measuring colony sizes in both single and double-mutant strains. SGAtools is a specialized tool designed for high-throughput analysis of colony growth, particularly useful in SGA studies [31]. The software computed the pixel count and normalized it against the average colony size on the plate, allowing for precise and unbiased quantitative assessments [32]. The experiment was replicated three times, and hits were designated as high-confidence if they exhibited a reduction of 30% or more in at least two repetitions. The data points that did not fall within the predetermined cutoff range or showed significant variance across the replicates were excluded [33]. Conditional SGA analysis was conducted by growing double deletion mutants on media supplemented with a mild sub-inhibitory concentration of cycloheximide to investigate gene interactions. Gene ontology enrichment tools were subsequently employed to categorize identified hits based on their biological processes and molecular functions. This tool utilizes Fisher’s Exact Test, leveraging the hypergeometric distribution, to calculate *p*-values and determine the statistical significance of the associations between specific GO terms and our chosen set of genes, as in [34]. In brief, this computation involves comparing the observed frequency of genes associated with each GO term against an expected frequency derived from the proportions found within a reference dataset (e.g., the entire yeast genome). The expected frequencies are based on the assumption of a random distribution of GO term associations across the genes in the reference set. The probability of observing a specified number of genes associated with a GO term within a chosen gene set is determined based on the following formula:

$$P\left(X=k\right)=\frac{\left(\begin{array}{c} K\\ k \end{array}\right)\cdot \left(\begin{array}{c} N-K\\ n-k \end{array}\right)}{\left(\begin{array}{c} N\\ n \end{array}\right)}$$

where *P* (*X* = *k*) is the likelihood of finding exactly *k* associated genes in our sample, *K* represents total number of associated genes in the reference set, *N* is the total number of genes in the reference dataset, *n* is our sample size, and *k* is identified genes that are associated with the GO term. The tool also complements the statistical analysis by considering the False Discovery Rate (FDR), which aims to control the expected proportion of false discoveries, ensuring the reliability of findings in indicating true biological significance [35].

In Phenotypic Suppression Array (PSA) analysis, a MATα yeast strain carrying an overexpression plasmid of our query gene is mated with the complete deletion mutant library array. An empty plasmid is also used as our reference for fitness control [36]. The constructs transformed into the deletion library were cultured on YPgal with a sub-inhibitory concentration of cycloheximide and compared to the control plasmid. This approach enabled us to assess the potential of overexpression of our query genes to offset the compromised phenotype of our deletion sets under the inhibitory concentration of cycloheximide. Consequently, a suggested functional association between these two genes could then be established [37]. The compensation analysis was repeated three times, and those that showed 30% or more compensation in at least two of the repeats were considered.

## 3. Results

### 3.1. Identification of YNR069C as a Potential Gene Involved in Protein Biosynthesis

We recently used a large-scale screening approach and identified certain candidate genes that, when deleted, the mutant strains showed an increase in the production of full-length proteins from an mRNA carrying a PTC [14]. To further study novel genes involved in stop codon bypass, we employed the plasmid, pUKC817, carrying a premature stop codon UAA within a *β-galactosidase* reporter gene and repeated the above screen. The plasmid was introduced into the yeast non-essential gene deletion array. Additionally, the control plasmid, pUKC815, carrying the native *β-galactosidase* gene (without PTC), was also systematically transformed into a deletion mutant array as a control. This systematic transformation was achieved through the adaptation of a large-scale mating approach initially designed for studying high-throughput genetic interactions in yeast [17].

**Figure 1:**
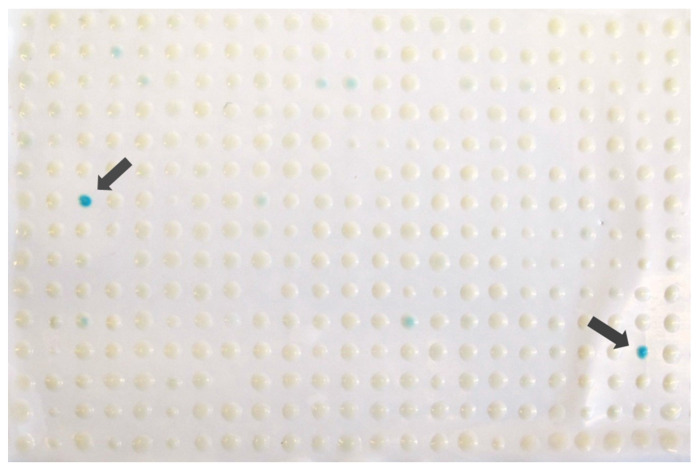
Colony lift assay for large scale screening of the mutant strains that affect PTC. Plasmid pUKC817 containing a UAA PTC in the *β-galactosidase* reporter gene was introduced into the yeast non-essential gene deletion array. The plasmid pUKC815, which contains no premature stop codons, was used as a control. The transformed yeast mutant colonies were subjected to a *β-galactosidase* lift assay. The blue colonies, indicated by arrows, represent the functional levels of *β-galactosidase* produced. This experiment was repeated three times.

First, the plasmid was introduced into the MATα strain. Subsequently, the transformed strains underwent systematic mating with a deletion library consisting of nearly 4500 mutants of MATa. The resulting diploid strains were then subjected to multiple rounds of selection to isolate haploid mutants carrying the plasmid with mutations in MATa. This process generates an array of strains, each bearing a specific gene deletion along with a target plasmid. To assess the translation activity of the *β-galactosidase* cassette in various mutants carrying target constructs, we conducted a *β-galactosidase* lift assay. In this assay, if the reporter gene is expressed and translated effectively, the cells would exhibit a blue color upon the addition of the X-gal solution, suggesting potential hits (Figure 1). White colonies, on the other hand, indicate mutant strains with inefficient translation of *β-galactosidase* mRNAs.

One of the genes that was not identified in our previous screen but now was consistently identified in different repeats was *YNR069C*, which codes for a protein of an unknown function. This observation underscores the limitation of large-scale screens in providing comprehensive insights, emphasizing the need for alternative approaches or repeated screenings to unveil new genes that may have been overlooked in the initial analyses. Notably, through a computational analysis, *YNR069C* was previously discovered to potentially carry a functional PTC, and hence, it was given the name *YNR069C* for Bypass of Stop Codon 5. The authors also reported that the rate of PTC bypass for this ORF is particularly high [38]. We selected this gene to further analyze its involvement in protein biosynthesis.

### 3.2. Deletion of YNR069C Is Linked to the Bypass of Premature Termination Codons

**Figure 2:**
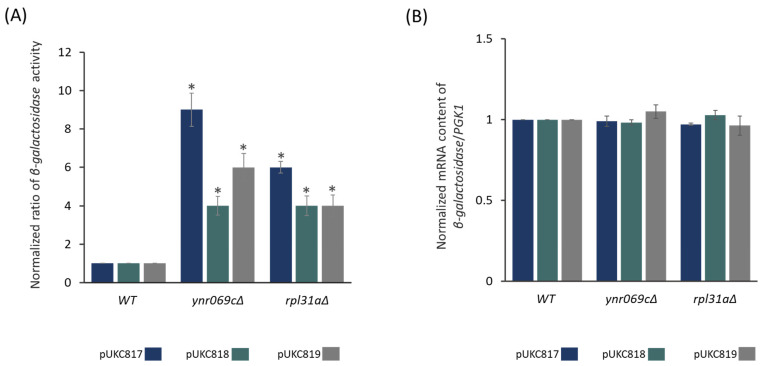
*β-galactosidase* expression analysis for *YNR069C* deletion mutant carrying the constructs containing premature stop codons. (**A**) The expression of *β-galactosidase* arising from constructs carrying premature stop codons (pUKC817, pUKC818, and pUKC819) is normalized to the *β-galactosidase* expression derived from pUKC815, which carries the native *β-galactosidase* gene and is compared to that of WT strain. (**B**) The levels of *β-galactosidase* mRNA are compared to those of the WT strain, with *PGK1* mRNA content used for normalization. Each data point represents the average generated from at least three independent experiments. The error bar represents the standard deviation. The asterisks represent statistically significant differences (ANOVA) from the WT (*p*-value ≤ 0.05).

To validate the accuracy of our high-throughput colony lift assays in identifying the *YNR069C* mutant strain with the capability of producing functional *β-galactosidase*, we subjected the mutant strains to a liquid ONPG-based quantitative *β*-galactosidase assay. As shown in Figure 2, deletion of *YNR069C* resulted in elevated levels of *β-galactosidase* expression across all three expression cassettes that carried different premature stop codons (UAA in pUKC817, UGA in pUKC818 and UAG in pUKC819). The values represented in Figure 2 are normalized to that of a control plasmid carrying no premature stop codon (pUKC815). This observed increase in *β-galactosidase* activity was comparable to that of the mutant strain for *RPL31A*, which was employed as a positive control.

Changes in mRNA content can also account for differences in *β-galactosidase* expression levels. To investigate this possibility, we analyzed the mRNA content of *β-galactosidase* in the gene deletion strains using qRT-PCR. No statistically significant differences were observed between the mRNA contents of the target and control strains (Figure 2B). This suggests that the observed increase in functional *β-galactosidase* expression is attributed to the translation process rather than being affected by changes in mRNA content.

### 3.3. Deletion of YNR069C Reduces the Rate of Protein Biosynthesis

**Figure 3:**
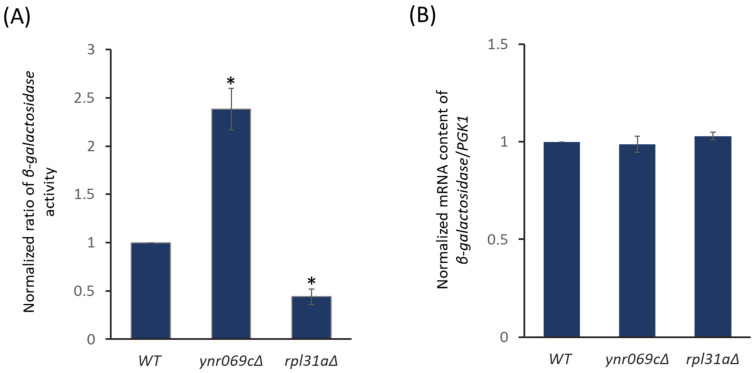
Evaluating the rate of protein synthesis in different yeast strains. (**A**) The translation rate was investigated by employing the *β-galactosidase* reporter expression cassettes, p416, that are under the transcriptional control of the inducible *GAL1* promoter. The obtained values are normalized to that of WT. (**B**) The deletion of *YNR069C* does not impact the mRNA content level for *β-galactosidase* mRNA. The housekeeping gene *PGK1* is employed as an internal control. Each data point is the average derived from at least three independent experiments, with error bars indicating the standard deviation. Asterisks denote statistically significant differences (ANOVA) from the WT with a *p*-value ≤ 0.05.

Next, we investigated whether the deletion of *YNR069C* had any influence on the translation rate by evaluating the expression of a native *β-galactosidase* using the p416 construct where *β-galactosidase* is under the transcriptional control of a *GAL1* inducible promoter. After the induction of *β-galactosidase,* we observed that, surprisingly, the deletion of *YNR069C* resulted in an increased level of protein biosynthesis, as shown in Figure 3A. However, the deletion of our control *RPL31A* decreased the level of translation as we expected. The *β-galactosidase* mRNA content remained consistent across all strains, indicating that the observed differences seem to be independent of transcription (Figure 3B).

### 3.4. Genetic Interaction Analysis Further Connects the Activity of YNR069C to Translation

For a deeper exploration of the roles of *YNR069C* in translation, we investigated its genetic interactions (GIs). Given that these interactions typically take place between functionally associated genes, examining the interaction network of the query genes could offer insights and predictions regarding their functions [39,40]. This phenomenon arises from functional redundancies, where the absence of one gene may not exhibit a noticeable effect on the phenotype, but when combined with the deletion of a second gene, it can lead to a different, unexpected phenotype [41]. Negative genetic interaction (nGI) implies that the simultaneous deletion of two genes produces a more severe phenotypic effect than the phenotypes observed with individual gene deletions alone [33]. An nGI between two genes suggests a functional association between the two gene functions and has been used as a measure to investigate the activity of various genes in DNA repair [42], cell cycle [43], transcription [26] and translation [39], etc.

Synthetic Genetic Array (SGA) analysis is a robust, high-throughput tool employed to explore nGIs in yeast [41]. The procedure involves the mechanical crossing of the query gene deletion strain of “α” mating type with an array of gene deletion strains of “a” mating type. Following several rounds of selection, double gene deletion mutant progenies are then chosen for further analysis [44]. The fitness of double gene deletion strains is then measured according to their colony size compared to that of single gene deletion strains (Supplementary Materials).

**Figure 4:**
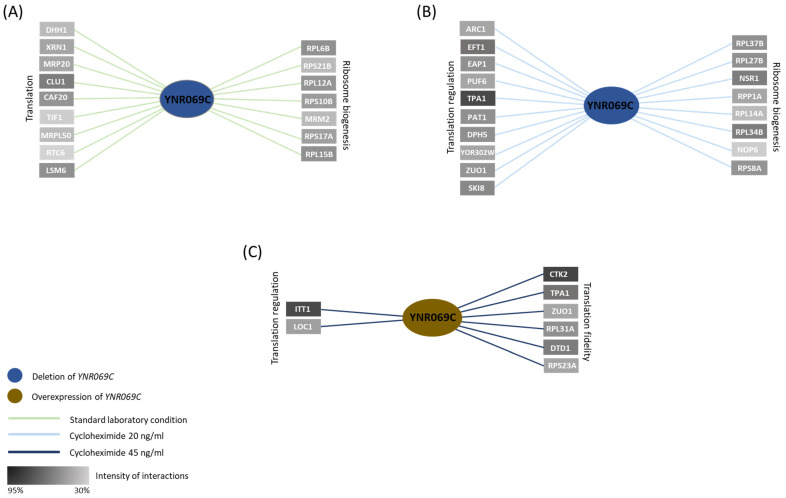
Genetic interaction analysis for *YNR069C*. (**A**) Under standard laboratory conditions, clusters of negative genetic interactions with genes involved in translation (*p*-value: 1.14 × 10^−10^) and ribosomal biogenesis (*p*-value: 3.77 × 10^−3^) were observed for *YNR069C*. (**B**) In the presence of a subinhibitory concentration of cycloheximide (20 ng/mL), a new set of interactors involved in translation regulation (*p*-value: 2.62 × 10^−11^) emerged. (**C**) Overexpression of *YNR069C* compensated for the cycloheximide (45 ng/mL) sensitivity of a group of genes that belongs to translation fidelity (*p*-value: 5.87 × 10^−5^). Color gradients represent the intensity of the interactions, with darker colors indicating a greater reduction in fitness. To ensure the reliability of these findings, only data from experiments with at least two repeats showing consistent results within a defined cut-off range were considered.

Examining the nGIs formed for *YNR069C* under standard laboratory conditions, we observed a number of interesting interactions. Clustering of these interacting genes based on Gene Ontology (GO) terms, we observed enrichment in two clusters, each associated with specific functional categories, ribosome biogenesis (*p*-value: 5.97 × 10^−8^), and translation process (*p*-value: 1.14 × 10^−10^) (Figure 4A). These observations further connect the activity of *YNR069C* to translation.

Under a mild sub-inhibitory concentration of cycloheximide (20 ng/mL), a compound that compromises translation in yeast, we observed the emergence of a novel set of interactions. Analysis of functional enrichment in the identified hits under this condition revealed a notable association between *YNR069C* and a range of genes involved in the translation regulation (*p*-value: 2.62 × 10^−11^) (Figure 4B). These observations suggest a possible new role for *YNR069C* in the regulation of translation only when translation is compromised.

Next, we investigated the capacity of *YNR069C* overexpression to reverse the phenotypes associated with various gene deletion mutants when exposed to cycloheximide treatment. Phenotypic Suppression Array (PSA) analysis is centered around a distinct type of interaction, wherein the overexpression of one gene serves to counterbalance the effects of the absence of another gene, particularly under the influence of a drug treatment [37]. First, we exposed the array of mutant strains to cycloheximide (45 ng/mL), resulting in heightened sensitivity in certain strains. Subsequently, we successfully reversed the sensitivity in several deletion mutants by introducing overexpression plasmids for *YNR069C.* The overexpression of *YNR069C* compensated the sensitivity to cycloheximide of deletion mutant strains for *CTK2*, *TPA1*, *ZUO1*, *RPL31A*, *DTD1*, *RPS23A*, and *RPS9B*, which are interestingly clustered under the category of regulation of translation fidelity (*p*-value: 5.87 × 10^−5^) (Figure 4C).

## 4. Discussion

Regulation of gene expression happens at various stages, including translation termination. The termination process of translation can also be impacted by the nucleotide sequence encompassing the stop codon. The occurrence of readthrough at regular stop codons is quite infrequent, but it can become notably higher if the genetic sequence context surrounding the stop codon favors this phenomenon [1,45]. Other mechanisms, such as ribosomal frameshifting and the influence of suppressor tRNAs, are also known to induce stop codon bypass [46].

Here, we observed that the deletion of *YNR069C* resulted in a substantial increase in the bypass of all three premature stop codons UAA, UAG, and UGA within a *β-galactosidase* reporter system, showing approximately 5, 6, and 10-fold enhancements, respectively. This level of stop codon readthrough is comparable to that observed for the *RPL31A* deletion mutant, which served as the positive control and showed enhancements of five-fold for both UAA and UAG PTCs and six-fold for UGA PTC. Interestingly, the deletion of *YNR069C* also led to a 2.5-fold increase in the rate of translation. In contrast, as expected, our positive control, *RPL31A*, showed a reduction in translation rate. qRT-PCR analysis demonstrated that the deletion of *YNR069C* did not exert any influence on the mRNA content of the *β-galactosidase* reporter constructs, indicating that the observed differences are independent of mRNA content levels. Based on the results obtained, deletion of *YNR069C* seems to enhance the protein biosynthesis rate but compromises the cell’s ability to recognize premature stop codons. Increasing the rate of protein synthesis may result in a lower accuracy of production. A possible interpretation of these findings is that *YNR069C* could be involved in preserving a balance between the quantity and quality of protein biosynthesis.

Building on these findings, the SGA analysis under both standard laboratory conditions and under mild sub-inhibitory concentration of cycloheximide revealed numerous intriguing interactions involving *YNR069C*, especially in the realm of translation and its regulation. These interactions were particularly noteworthy in the context of translation and translation control, suggesting a function for *YNR069C* within the process of translation. Additionally, our PSA analysis provided deeper insights. Overexpression of *YNR069C* was found to counteract the phenotypes associated with various gene deletion mutants under cycloheximide treatment. Gene Ontology (GO) clustering revealed significant enrichment in clusters associated with translation fidelity, supporting a link between *YNR069C* activity and translation fidelity.

Specifically, the observed GIs with genes such as *CTK2*, *TPA1*, *ZUO1*, *RPL31A*, *DTD1*, *RPS23A*, and *RPS9B*, which clustered under the translation fidelity category, were noteworthy. *CTK2*, a subunit of complex CTDK-I, is involved in translation by phosphorylating *RPS2*, a component of the small ribosomal subunit. This phosphorylation event enhances the decoding fidelity during translation elongation. A reduction in the levels of *CTK2* leads to a defect in translation elongation, manifested by an increased frequency of miscoding [47]. *TPA1* (termination and polyadenylation factor) plays a role in translation termination accuracy. Yeast strains with a deletion of the *TPA1* displayed enhanced readthrough of stop codons. Coimmunoprecipitation assays further indicated that Tpa1p interacts with the translation termination factors eRF1 and eRF3 [48,49]. *ZUO1* is another interactor that encodes a ribosome-associated chaperone. The localization of *ZUO1* near the 60S polypeptide-exit site indicates potential interactions with the ribosomal protein eL31. Moreover, *ZUO1* also engages with 40S ribosomes. Its dual interaction with both the 40S and 60S ribosomal subunits suggests a potential role in protein quality control mechanisms. Notably, deletions in the C terminus of *ZUO1* lead to altered readthrough of stop codons [50,51]. *RPL31A* is another interacting gene that shows high sensitivity to aminoglycoside antibiotics, and the cells experience defects in translational fidelity when deleted [52]. *DTD1* has been reported to impact nonsense suppression likely by affecting tRNA-mediated nonsense suppression [53]. *RPS23A* encodes ribosomal proteins 28 of the 40S subunit and is shown to affect translational accuracy [54,55]. Furthermore, we identified other notable interactors, including *ITT1* (Figure 4C), which interact with the translation release factor eRF3 and have a significant impact on the efficiency of translation termination.

The increased PTC bypass observed in the absence of *YNR069C* suggests a regulatory function for this gene within the translation process, which has been supported by our genetic interaction analysis. This finding is particularly noteworthy because PTC bypass can have profound implications for both normal cellular functioning and disease state, where a nonsense mutation can lead to aberrant protein synthesis. Conditions such as Duchenne Muscular Dystrophy (DMD) [56,57], Cystic Fibrosis [58], Beta-Thalassemia [59], Spinal Muscular Atrophy (SMA) [60], Hurler Syndrome [61], Ataxia Telangiectasia [62], and certain form of cancer [63] are prime examples. In these diseases, PTCs lead to the synthesis of truncated, typically non-functional proteins.

Therapies that can induce ribosomes to read through these premature stop codons offer a potential treatment strategy to restore at least partial function of the affected protein. By understanding how *YNR069C* influences PTC bypass, we can potentially uncover new therapeutic approaches for diseases where the restoration of the full-length, functional protein is crucial. This highlights the broader relevance of our findings in the field of medical genetics and underscores the potential impact of our research in developing treatments for diseases caused by nonsense mutations.

Additionally, there are scenarios where PTC bypass plays a different role tied to the regulation of gene expression itself. *YNR069C* and its adjacent ORF *YNR068C* are separated by a stop codon. This stop codon can be bypassed in translation, allowing for the production of a larger protein referred to as Bul3p. *BUL3* is reported to play a role in ubiquitin-mediated sorting of plasma membrane proteins via Rsp5p [15]. This instance of PTC bypass is not just a matter of correcting a genetic error; rather, it appears to be a sophisticated regulatory mechanism. In this context, *YNR069C* may have a positive role in regulating the translation of its own mRNA, preventing needless stop codon readthrough allowing cells to adaptively control the production of Bul3p based on cellular needs.

In future studies, we aim to investigate the expression and regulation of *YNR069C* in response to different environmental and physiological stimuli. This will include analyses under various growth conditions, such as exposure to different stress factors, nutrient availability, and during different growth phases. By doing so, we anticipate uncovering further insights into the functional dynamics of *YNR069C*, potentially revealing how this protein modulates translation fidelity in response to the cellular environment and thereby broadening the impact and applicability of our research.

## 5. Conclusions

In conclusion, our investigation into gene expression regulation, with a particular focus on translation termination and fidelity, has revealed the intriguing involvement of the *YNR069C* gene. The observed increase in PTC bypass and enhanced translation rate upon *YNR069C* deletion strongly suggest its role in modulating translation accuracy and efficiency.

Moreover, genetic interaction analysis provides compelling support for our hypothesis, associating *YNR069C* with the regulation of translation. This finding adds a valuable piece to the puzzle of understanding the nuanced mechanisms governing gene expression and protein synthesis. Further exploration of *YNR069C* and its interplay with translation processes holds promise for unraveling novel aspects of gene regulation.

## Data Availability

All data generated and/or analyzed during this study are included in this research article and/or its Supplementary Material Files.

## Supplementary Materials

The following supporting information can be downloaded at https://www.mdpi.com/article/10.3390/biology13030138/s1: List of genes identified in SGA, conditional SGA, and PSA, with their normalized growth scores from three replicates.
